# Clinical and prognostic significance of various anemia types in pulmonary arterial hypertension

**DOI:** 10.3389/fmed.2025.1728165

**Published:** 2025-12-10

**Authors:** Marcin Waligóra, Maria Mrowicka, Miłosz Pietrus, Justyna Daniek, Patrycja Kurczyna, Michał Karnaś, Weronika Chaba-Karnaś, Kamil Jonas, Grzegorz Kopeć

**Affiliations:** 1 Pulmonary Circulation Centre Department of Cardiac and Vascular Diseases, Jagiellonian University Medical College, Krakow, Poland; 2 Department of Cardiac and Vascular Diseases, St. John Paul II Hospital, Krakow, Poland; 3 Department of Medical Education, Faculty of Medicine, Center for Innovative Medical Education, Jagiellonian University Medical College, Krakow, Poland; 4 Department of Gynecology and Oncology, Faculty of Medicine, Jagiellonian University Medical College, Krakow, Poland

**Keywords:** pulmonary hypertension, anemia, prognosis, idiopathic pulmonary hypertension, risk stratification

## Abstract

**Introduction:**

Current risk models for pulmonary arterial hypertension (PAH) omit anemia, although it may aggravate symptoms and haemodynamics. We assessed whether anemia and its subtypes add prognostic value.

**Methods:**

This study included patients with idiopathic (IPAH) or connective tissue disease-associated PAH (CTD-PAH). Anemia was classified per WHO criteria into four subtypes: anemia of chronic disease (ACD), iron deficiency anemia (IDA), ACD/IDA overlap, and multifactorial anemia. Risk stratification followed the ESC 2022 guidelines and was reassessed after 12 months.

**Results:**

Among 127 patients (78.7% IPAH, 21.3% CTD-PAH) anemia was present in 22 patients (17.3%), including ACD (31.8%), ACD/IDA overlap (27.3%), and multifactorial anemia (40.9%). Patients with anemia had higher baseline risk strata, worse functional status, elevated NT-proBNP levels (*p* = 0.002), and shorter six-minute walk distances (*p* = 0.007). Anemia reduced the odds of clinical improvement by 75% (OR, 0.25; *p* = 0.02). Over a follow-up of 57.8 months, 64 patients (50%) died. ACD (HR, 2.43; 95% CI, 1.03–5.7; *p* = 0.04) and ACD/IDA (HR, 2.14; 95% CI, 1.04–4.41; *p* = 0.04) independently predicted mortality, even after adjustment for age and risk.

**Conclusion:**

Anemia, particularly ACD and ACD/IDA, independently predicts a lower probability of achieving therapeutic goals within 12 months and is associated with higher long-term mortality, even after adjustment for the ESC/ERS 2022 recommended risk stratification.

## 1 Introduction

Pulmonary arterial hypertension (PAH) is a progressive disease characterized by elevated pulmonary vascular resistance and right ventricular failure. In Poland, it affects up to 30.8 adults per million with the annual incidence of 5.2 per million (1). The 2022 guidelines of the European Society of Cardiology and the European Respiratory Society (ESC/ERS) highlight a multifaceted risk stratification approach for PAH, incorporating clinical, hemodynamic, laboratory, and imaging parameters to guide management and predict outcomes. Despite advances in PAH treatment (2–4), the condition remains associated with high mortality (5, 6). Among laboratory variables, iron deficiency and anemia have gained attention as potential prognostic factors, although they are not yet fully incorporated into routine risk stratification models (7).

Anemia and iron deficiency are common comorbidities in PAH and have important implications for clinical outcomes. Iron deficiency has been linked to impaired exercise capacity, reduced quality of life, and increased mortality in patients with PAH, even in the absence of anemia (8, 9). The ESC/ERS guidelines recommend routine monitoring of iron parameters, including serum iron, ferritin, and transferrin saturation (TSAT). However, the specific effects of different anemia subtypes, such as anemia of inflammation (known also as anemia of chronic disease [ACD]) or the overlap between ACD and iron deficiency anemia (IDA), on clinical improvement and mortality in PAH remain insufficiently studied. Moreover, it remains unclear whether iron deficiency is a causal factor in poor outcomes or merely a marker of disease severity. Importantly, the presence of anemia may exacerbate dyspnoea and affect haemodynamics (10), potentially influencing the accuracy of risk stratification.

This study aims to evaluate the prevalence and prognostic significance of various types of anemia and deficiencies in iron, vitamin B12, and folate in patients with PAH, with a particular focus on their association with clinical risk and mortality, independent of established risk factors.

## 2 Materials and methods

### 2.1 Study population

The study included adult patients recruited consecutively from the Pulmonary Vascular Disease Programme at the St. John Paul II Hospital in Krakow, Poland between 2009 and 2022. Patients were eligible if diagnosed with idiopathic PAH (IPAH) or PAH associated with connective tissue disease (CTD-PAH) and fulfilled the haemodynamic definition of PAH valid at the time (mPAP ≥ 25 mmHg). Patients with other forms of PAH, chronic thromboembolic disease, significant left heart disease, or pulmonary disease were excluded as presented in flowchart (Supplementary Figure 1).

The study protocol was approved by the local bioethics committee and conducted in accordance with the principles of the Declaration of Helsinki. Informed consent was obtained from all participants prior to enrolment. The institutional ethics committee approved the registry of patients with PAH and CTEPH in our center (66/KBIL/OIL/2009), followed by subsequent approval for analyses (102.6120.272.2022).

### 2.2 Right heart catheterization

Right heart catheterization was performed with the patient in the supine position, using access via the right femoral vein or the right internal jugular vein, and a Swan–Ganz catheter. Pressure waveforms were recorded at end-expiration during normal breathing. Cardiac output was measured using the Fick method with direct measurement of oxygen consumption. Cardiac index was calculated by dividing cardiac output by body surface area, which was determined using the Mosteller formula (11). Pulmonary vascular resistance was calculated as the difference between mean pulmonary arterial pressure and pulmonary artery wedge pressure, divided by cardiac output.

### 2.3 Iron metabolism, anemia classification, and nutrient absorption impairments

Blood samples were collected, centrifuged, and plasma was stored at −80 °C until analysis. The following parameters were measured: ferritin, iron, total iron-binding capacity, unsaturated iron-binding capacity, TSAT, as well as folic acid and vitamin B12 concentrations. All those measurements were performed in 2023 in a single certified laboratory, using the same analytical platform and identical assay methods for all samples.

Iron deficiency (ID) was defined as a serum ferritin level of less than 100 μg/L or between 100 and 299 μg/L and TSAT below 20% (7).

Dysutilization of iron for erythropoiesis was identified in patients with normal hemoglobin levels but laboratory features consistent with ACD. This condition was defined as TSAT of less than 20% with ferritin concentration greater than 100 μg/L, irrespective of hemoglobin levels.

Anemia was defined based on the World Health Organization (WHO) criteria as a hemoglobin level of less than 13 g/dL in men and less than 12.0 g/dL in women (12). Patients with anemia were further classified into the following subtypes based on established definitions (13, 14):

- ACD (also known as anemia of inflammation): TSAT < 20%, ferritin > 100 μg/L (14).
- IDA: TSAT < 20%, ferritin < 30 μg/L.
- ACD/IDA overlap: TSAT < 20%, ferritin 30–100 μg/L.
- Multifactorial anemia: TSAT > 20%, not meeting the criteria for other anemia subtypes.

Data on comorbidities that could impair nutrient absorption were also collected. These included celiac disease, Crohn disease, chronic gastritis, Helicobacter pylori infection, status post ileal resection, status post gastrectomy, chronic pancreatitis, pancreatic cancer, cystic fibrosis, gallstones, and cholangitis.

### 2.4 Risk assessment and stratification

Baseline risk was assessed using the three-strata risk stratification model as recommended in the 2022 ESC/ERS guidelines (7). For follow-up evaluations, the COMPERA 2.0 four-strata model was applied (15). This model integrates WHO functional class, six-minute walking distance (6MWD), and N-terminal pro–brain natriuretic peptide (NT-proBNP) levels to categorize patients into four risk groups: low, intermediate-low, intermediate-high, or high mortality risk.

Clinical improvement was evaluated by comparing four-strata mortality risk at diagnosis and after 12 months of follow-up. Patients were classified as improvers if they demonstrated a decrease of at least one risk category (e.g., from intermediate-high to intermediate-low risk) or achieved low-risk status. Those whose disease did not improve or worsened or who died before the 12-month follow-up were classified as non-improvers.

### 2.5 Statistical analysis

Continuous variables were presented as medians with interquartile ranges (IQRs) due to non-normal distribution, as determined by the Shapiro–Wilk test. Categorical variables were presented as counts and percentages. Group comparisons (e.g., between patients with and without anemia) were performed using the Mann–Whitney test for continuous variables and the chi-square test or Fisher exact test for categorical variables, as appropriate. For small sample sizes, Yates correction was applied to the chi-square test.

Logistic regression was used to evaluate associations between clinical improvement at one year (defined in the “2.4 Risk assessment and stratification” section above) as the dependent variable and anemia subtypes or iron deficiency as independent variables. Results are reported as odds ratios (OR) and 95% confidence intervals (CI).

Cox proportional hazards regression was used to identify predictors of mortality. Significant predictors were subsequently included in multivariate Cox regression models. All multivariable Cox regression models included adjustment for baseline ESC/ERS mortality risk classification [three-strata model: low, intermediate, high or COMPERA 2.0 four-strata model (15): low, intermediate-low, intermediate-high, high measured as change per 1 strata category (negative/positive)] and age (7). Results are presented as hazard ratios (HR) with 95% CIs.

Kaplan–Meier survival curves were generated to visualize differences in survival, with comparisons made using the log-rank test. Correlations between continuous variables were assessed using Spearman’s rank correlation coefficient (ρ). A two-sided *p*-value < 0.05 was considered statistically significant. All analyses were performed using the Dell Statistica data analysis software, version 13.3 (TIBCO Software Inc., Palo Alto, California, United States) and the MedCalc software, version 19.2.6 (MedCalc Software, Ostend, Belgium).

## 3 Results

### 3.1 Study characteristics

The study cohort included 127 patients with PAH (37 men, 90 women) with a median age of 57 years (IQR, 45–69). Of these, 100 patients (78.7%) had IPAH and 27 (21.3%) had CTD-PAH. The detailed flowchart illustrating the process of patient inclusion and exclusion is depicted in Supplementary Figure 1. Blood samples were collected at the baseline visit for most patients (*n* = 93, 73.2%) and during follow-up for the remainder. As shown in Table 1, the majority were in WHO functional class III or IV and presented severe hemodynamic impairment. Most patients were classified as intermediate risk (*n* = 99, 78%), followed by low risk (*n* = 14, 11%) and high risk (*n* = 14, 11%).

**TABLE 1:** Characteristics of the study population by anemia status.

Parameter	Whole population (*n* = 127)	Anemia (*n* = 22)	No anemia (*n* = 105)	*p*-value
**Diagnosis**				
IPAH	100 (78.7)	16 (72.7)	84 (80)	0.45
CTD-PAH	27 (21.3)	6 (27.3)	21 (20)	
Incident cases	93 (73.7)	19 (86.4)	74 (79.6)	0.13
Female sex	90 (70.9)	11 (50)	78 (74.3)	0.02
Age (y)	57 (45–69)	69 (67–73)	54 (43–68)	0.002
eGFR (mL/min/1.73 m^2^)	71 (55–89)	49 (39–74)	72.5 (59–93)	0.002
Creatinine (mg/dL)	0.85 (0.83–1.12)	1.11 (0.87–1.64)	0.95 (0.83–1.11)	0.028
hsCRP (mg/L)	3 (1.45–6.94)	6.38 (2–26)	2.85 (1.44–5.16)	0.06
IL-6 (pg/mL)	16.5 (9.8–28.2)	15.3 (8.9–29.7)	16.7 (9.8–27.3)	0.86
WHO-FC I or II	16 (12.6)	2 (9.1)	14 (13.3)	0.85^⊛^
WHO-FC III or IV	111 (87.4)	20 (90.1)	91 (86.7)	
NT-proBNP (pg/mL)	1,431 (488–3,057)	2,799 (1,354–8,588)	1,204 (461–2,642)	0.002
6MWD (m)	310 (210–390)	210 (135–357)	315 (240–400)	0.007
mPAP (mmHg)	44.5 (37–53)	40.5 (37–49)	45.5 (37–54)	0.18
mRAP (mmHg)	6 (3–9)	8.5 (6–13)	5 (3–7)	0.005
Cardiac index (L/min/m^2^)	2 (1.7–2.4)	2.3 (2–2.8)	2 (1.7–2.4)	0.02
PVR (WU)	9.9 (7.1–14.8)	7.4 (5.3–11.1)	10.4 (7.3–14.9)	0.02
TAPSE (mm)	18 (15–21)	15.5 (12–22)	18 (15–21)	0.24
RAA (cm^2^)	26 (22–34)	30.5 (24–35)	25.8 (21–33.5)	0.07
More than minimal pericardial effusion	9 (7.1)	5 (22.7)	4 (3.8)	0.02^⊛^
**Baseline clinical risk in PAH**				
Low	14 (11)	2 (9.1)	12 (11.4)	0.49
Intermediate	99 (78)	16 (72.7)	83 (79.1)	
High	14 (11)	4 (18.2)	10 (9.5)	
Improved clinical status within year	53 (41.7)	4 (18.2)	49 (46.7)	0.014^⊛^
**PAH specific therapy at the time of baseline assessment**				
Reactive PAH treated with calcium channel blocker	6 (4.7%)	0	6 (5.7%)	0.55^⊛^
**Non-reactive PAH**				
PDE5i	22 (17.3%)	1 (4.5%)	21 (20%)	0.15^⊛^
Endothelin receptor antagonist	11 (8.7%)	1 (4.5%)	10 (9.5%)	0.74^⊛^
Prostacyclin analogs	10 (7.9%)	2 (9.1%)	8 (7.6%)	0.84^⊛^
PAH specific therapy within 12 months*				
Reactive PAH treated with calcium channel blocker	9 (7.1%)	0	9 (8.6%)	0.33^⊛^
**Non-reactive PAH**				
PDE5i	95 (74.8%)	18 (81.8%)	78 (74.2%)	0.63^⊛^
Endothelin receptor antagonist	51 (40.2%)	8 (36.4%)	43 (41%)	0.69
Prostacyclin analogs	58 (%)	7 (31.8%)	51 (48.6%)	0.15

⊛ Yates correction was applied.

*available for 109 patients who survived first 12 months (17 with anemia and 92 without anemia). Data are presented as *n* (%) or median (IQR). 6MWD, 6-min walking distance; CTD-PAH, connective tissue disease–associated pulmonary arterial hypertension; eGFR, estimated glomerular filtration rate; hsCRP, high-sensitivity C-reactive protein; IL-6, interleukin-6; IPAH, idiopathic pulmonary arterial hypertension; mPAP, mean pulmonary arterial pressure; mRAP, mean right atrial pressure; NT-proBNP, N-terminal pro–brain natriuretic peptide; PAH, pulmonary arterial hypertension; PVR, pulmonary vascular resistance; RAA, right atrial area; TAPSE, tricuspid annular plane systolic excursion; WHO-FC, World Health Organization functional class.

**FIGURE 1:**
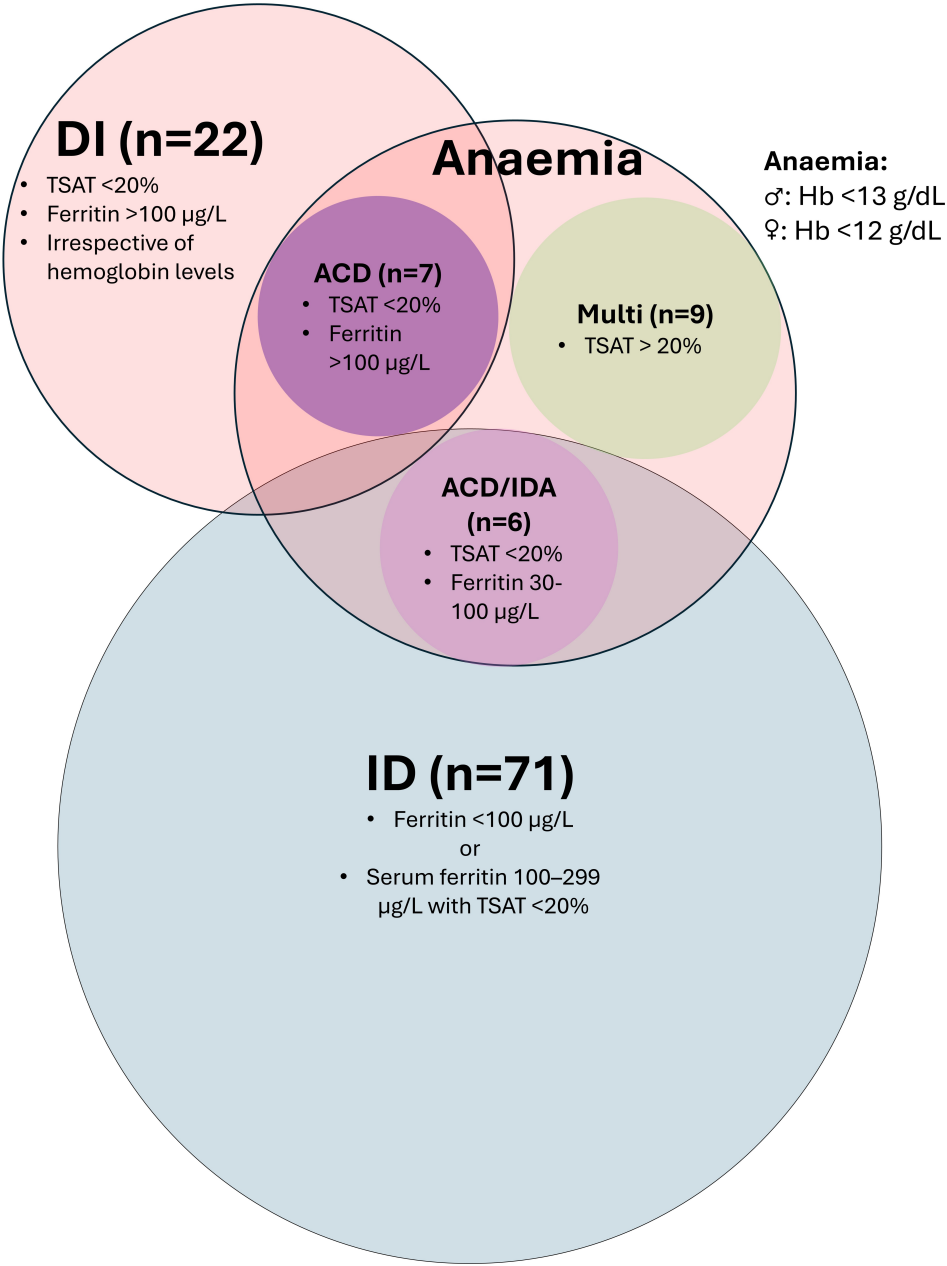
Visual representation of classification of iron metabolism disturbances and anemia subtypes. ACD, anemia of chronic disease; ACD/IDA, overlap of ACD and IDA; DI, dysutilization of iron; ID: iron deficiency; IDA, iron deficiency anemia; Multi, multifactorial anemia; TSAT, transferrin saturation.

Anemia was identified in 22 patients (17.3%) and classified as ACD in 7 patients (31.8%), ACD/IDA overlap in 6 patients (27.3%), and multifactorial anemia in 9 patients (40.9%). Notably, no cases of isolated IDA were observed. Iron deficiency was present in 71 patients (55.9%), and dysutilization of iron for erythropoiesis was observed in 22 patients (17.3%). An overview of anemia subtypes and iron metabolism abnormalities is shown in Figure 1.

### 3.2 Patient characteristics according to anemia status

Patients with anemia were significantly older, were more likely to be male, and had lower estimated glomerular filtration rate compared with those without anemia. Patients with anemia demonstrated worse clinical and functional parameters. They had significantly higher levels of NT-proBNP and shorter 6MWD. Hemodynamic assessments showed that those with anemia had higher mean right atrial pressure and cardiac index and lower pulmonary vascular resistance. Baseline mortality risk was similar in patients with and without anemia. Background PAH-specific therapy (monotherapy, dual, or triple therapy) at the time of sample collection did not differ between anemic and non-anemic patients (9.1%, 4.5%, 0% vs. 12.3%, 9.5%, 1.9%, respectively; *p* = 0.72). At the 12-month assessment, therapy intensity also remained comparable (63.6%, 22.7%, 13.6% vs. 32.4%, 38.1%, 20.0%; *p* = 0.06). Detailed data are presented in Table 1, and an expanded characterization of anemia subgroups is provided in the Supplementary results.

### 3.3 Different types of anemia and deficiencies across risk categories

**FIGURE 2:**
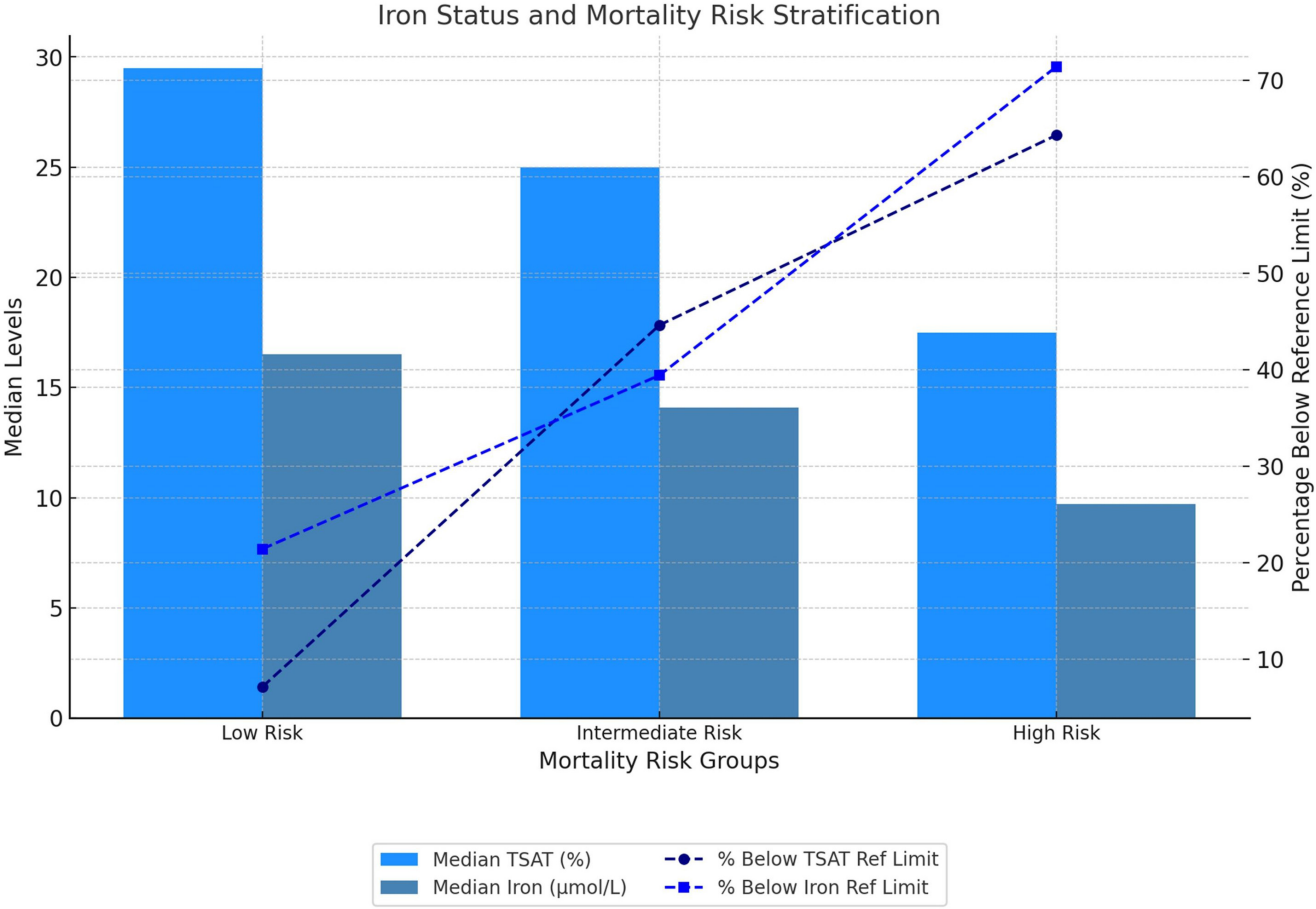
Iron status and baseline mortality risk stratification. Mortality risk was stratified at baseline according to the three-strata risk model recommended by the European Society of Cardiology/European Respiratory Society (ESC/ERS) guidelines. TSAT, transferrin saturation.

The prevalence of ACD varied across risk categories, being the highest in the high-risk group (21.4%), compared to the intermediate-risk (4%) and the low-risk (0) groups (*p* = 0.02). Similarly, iron deficiency was more prevalent in the high-risk group (50%) than in the intermediate-risk (34.4%) and low-risk (7.1%) groups (*p* = 0.047). Median TSAT demonstrated a decreasing trend across risk groups: 29.5% (IQR, 21–36) in the low-risk group to 25% (IQR, 15–33) in the intermediate-risk group, and 17.5% (IQR, 11–22) in the high-risk group (*p* = 0.005). The proportion of patients with TSAT below the lower reference limit increased, from 7.1% in the low-risk group to 44.6% in the intermediate-risk group and 64.3% in the high-risk group (*p* = 0.007). A similar pattern was observed for serum iron concentrations, which decreased from 16.5 μmol/L (IQR, 13.3–21.9) in the low-risk group to 14.1 μmol/L (IQR, 8.6–20) in the intermediate-risk group and 9.7 μmol/L (IQR, 7.6–11.1) in the high-risk group (*p* = 0.01). The proportion of patients with serum iron levels below the lower reference limit also increased across risk strata: 21.4% in the low-risk group, 39.4% in the intermediate-risk group, and 71.4% in the high-risk group (*p* = 0.02). The results are visually summarized in Figure 2.

Vitamin B12 and folic acid deficiencies were uncommon in our cohort. Detailed characteristics and comparisons between risk strata are provided in the Supplementary material.

### 3.4 Prognostic role of anemia

**FIGURE 3:**
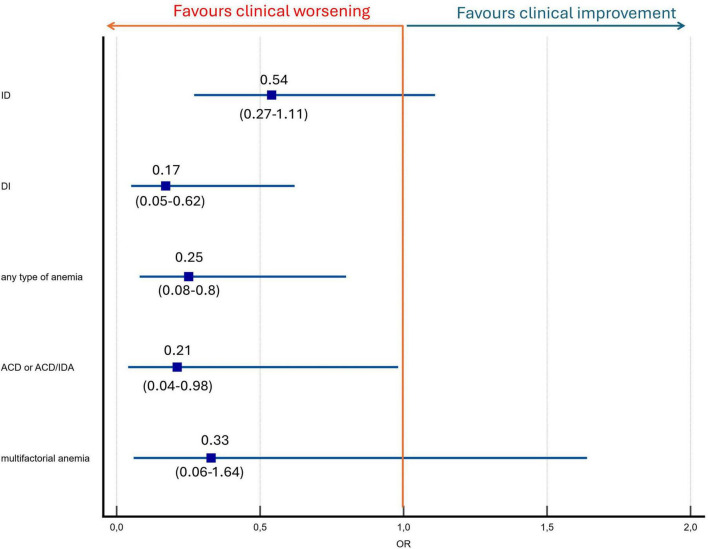
Anemia and iron status predictors of clinical improvement by at least one class in four-strata risk stratification model or reaching low risk status. ACD, anemia of chronic disease; ACD/IDA, ACD and IDA overlap; DI: dysutilization of iron; ID, iron deficiency.

At the one-year follow-up, patients with anemia at baseline were significantly less likely to achieve clinical improvement compared to those without anemia (18.2% vs. 46.7%; *p* = 0.01). Dysutilization of iron at baseline was more prevalent among non-improvers than improvers (25.7% vs. 5.7%; *p* = 0.003) (Table 2). In a logistic regression model, anemia was significantly associated with a reduced likelihood of clinical improvement. Patients with anemia had four-fold lower odds for improvement by at least one class or by reaching low-risk status profile (OR, 0.25; 95% CI, 0.08–0.8; *p* = 0.02). The impact of different types of anemia on the likelihood of clinical improvement is presented in Figure 3. Within the first 12 months after baseline blood sampling, there were no differences in PAH-specific therapy between groups (Table 1). Supplementation with iron, folic acid, or vitamin B12 was not associated with clinical improvement (all *p* > 0.1). PAH-specific therapy at baseline did not differ between improvers and non-improvers (9.4%, 7.5%, 0% vs. 13.5%, 9.5%, 2.7%, respectively; *p* = 0.59). At the 12-month assessment, treatment intensity also remained comparable between the two groups (32.1%, 32.1%, 24.5% vs. 41.9%, 37.8%, 14.9%, respectively; *p* = 0.28).

**TABLE 2:** Comparison of clinical and laboratory parameters by improvement in clinical status at 12 months.

Parameter	No improvement in clinical status within 12 months (*n* = 74)*	Improvement in clinical status within 12 months (*n* = 53)	*p*-value
**Quantitative parameters**			
Dysutilization of iron	19 (25.7)	3 (5.7)	0.003
Iron deficiency	46 (62.2)	25 (47.2)	0.09
**Anemia**			
Any type of anemia	18 (24.3)	4 (7.6)	0.02**
ACD	7 (9.5)	0	0.04 **
ACD/IDA overlap	4 (5.1)	2 (3.8)	0.8**
ACD or ACD/IDA overlap	11 (14.9)	2 (3.8)	0.03**
Multifactorial anemia	7 (9.5)	2 (3.8)	0.21**
Isolated IDA	0	0	–
**Folic acid and vitamin B12**			
Folic acid (ng/mL)	5.0 (3.3–7.5)	4.8 (3.9–7.3)	0.65
Folic acid < LRL	24 (32.4)	11 (20.8)	0.15
B12 (pg/mL)	460.5 (367–590)	438 (309–630)	0.49
B12 < LRL	1 (1.4)	4 (7.6)	0.08
**Iron metabolism parameters**			
TIBC (μmol/L)	60.2 (50–67.8)	60.3 (53.1–68.2)	0.48
TIBC < LRL	9 (12.2)	3 (5.7)	0.22
TIBC > URL	13 (17.6)	13 (24.5)	0.34
UIBC (μmol/L)	43.7 (34.8–55.2)	45.2 (35.8–54)	0.96
UIBC < LRL	2 (2.7)	3 (5.7)	0.4
UIBC > URL	8 (10.8)	6 (11.3)	0.93
Transferrin saturation (%)	21 (15–31)	27 (17–35)	0.15
Transferrin saturation < LRL	36 (48.7)	18 (34)	0.1
Iron (μmol/L)	11.9 (8.4–19.2)	15.6 (9.5–20.5)	0.11
Iron < LRL	36 (48.7)	16 (30.2)	0.04
Ferritin (μg/L)	104.1 (70.6–195.1)	109.8 (52.1–254.7)	0.95
Ferritin < LRL	4 (5.4)	7 (13.2)	0.12
Ferritin > URL	5 (6.8)	5 (9.4)	0.58

Data are presented as *n* (%) or median (IQR). ACD, anemia of chronic disease; ACD/IDA, overlap of ACD and IDA; IDA, iron deficiency anemia; LRL, lower reference limit; TIBC, total iron-binding capacity; UIBC, unsaturated iron binding capacity; URL, upper reference limit. To convert folic acid to nmol/L, multiply by 2.266; B12 to pmol/L, by 0.7378; iron to μmol/L, by 0.179; ferritin to μg/L, by 1.0. *worsening or no change in 4 strata risk assessment or death;

**Yates correction was applied. *P*-values were calculated for comparisons between each anemia subtype and patients without anemia.

**FIGURE 4:**
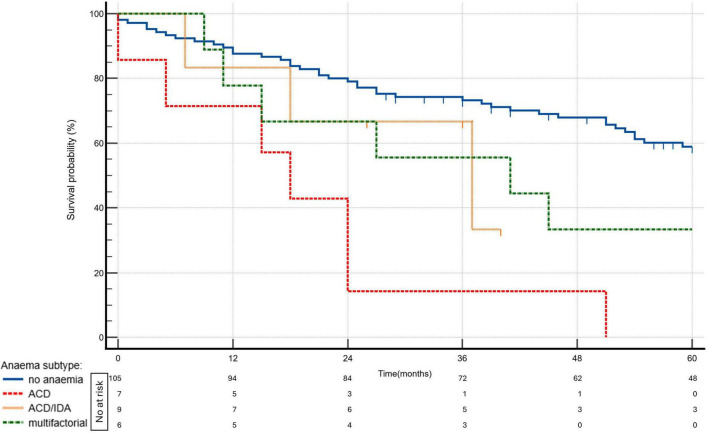
Kaplan—Meier survival curves by anemia status and subtypes (*p* = 0.0001). ACD, anemia of chronic disease; ACD/IDA, overlap of ACD and IDA; IDA, iron deficiency ananemia.

Over a median follow-up of 51 (24–86) months, 64 patients (50%) died. Kaplan–Meier survival analysis demonstrated significant differences in survival between patients without anemia and those with various types of anemia. The survival curves, shown in Figure 4, highlight the distinct impact of anemia subtypes on long-term outcomes.

The univariate analysis demonstrated that the presence of dysutilization of iron was associated with a two-fold higher risk of mortality (HR, 2.01; 95% CI, 1.15–3.5; *p* = 0.01). ACD and ACD/IDA overlap were even stronger predictors of mortality. ACD was associated with a 5.36-fold increased risk (HR, 5.36; 95% CI, 2.34–12.3; *p* < 0.0001) and ACD/IDA overlap was associated with a 3.62-fold increased risk (HR 3.62, 95% CI: 1.77–7.43, *p* = 0.0004), all compared to patients without anemia.

**FIGURE 5:**
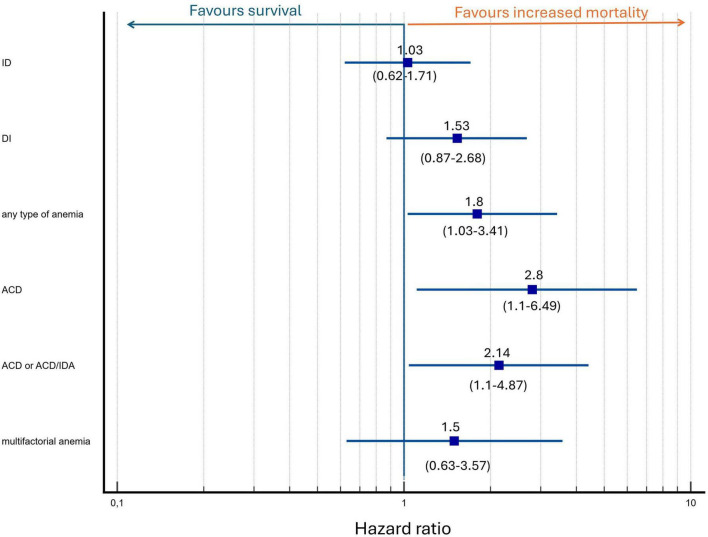
Multivariate analysis of anemia and iron status as predictors of mortality adjusted for baseline mortality risk and age. Hazard ratio depicted as logarithmic scale.

When adjusted for age and the three-strata risk score, the presence of any type of anemia or ACD remained a significant predictor of mortality (HR, 1.88; 95% CI, 1.03–3.42; *p* = 0.04 and HR, 2.68; 95% CI, 1.11–6.49; *p* = 0.03, respectively). Anemia with TSAT < 20% regardless of ferritin concentration (ACD or ACD/IDA overlap combined) may be associated with increased mortality (HR, 2.14; 95% CI, 1.04–4.41; *p* = 0.04); however, given the limited number of patients in these subgroups, these findings should be interpreted with caution. The results are depicted in Figure 5. The three-strata risk score consistently demonstrated a strong predictive value in these models and age remained a significant contributor to mortality risk, as presented in Table 3. To assess whether anemia predicts mortality independently of dynamic changes in clinical risk, we performed an expanded multivariable Cox model incorporating the change in the COMPERA 2.0 four-strata mortality category between baseline and 12 months (Δ 4-strata; available only for patients who survived to the 12-month reassessment). In this model, anemia remained a significant and independent predictor of mortality (HR 1.95, 95% CI 1.00–3.80; *p* = 0.048), together with Δ 4-strata change. The results are presented in Table 3, model 2.

**TABLE 3:** Multivariate Cox regression analyses.

Model of risk stratification	HR	95% CI	*p*-value
**First model: 3 strata mortality risk, age, any type of anemia**			
3 strata risk category	3.35	1.82–6.18	0.0001
Anemia of any type	1.88	1.03–3.42	0.04
Age [per 1 year]	1.03	1.01–1.05	0.0006
Second model*: 3 strata mortality risk, change in 4 age, any type of anemia, change in 4-strata mortality risk category			
3 strata risk category	5.55	2.56–12	< 0.0001
Δ 4-strata category change	1.94	1.35–2.81	0.0004
Anemia of any type	1.95	1.005–3.8	0.048
Age [per 1 year]	1.03	1.007–1.05	0.008
**Second model: 3 strata risk, age, any type of anemia, use of PAH specific drug (mono, duo or triple combination therapy)**			
3 strata risk category	3.4	1.81–6.38	0.0001
Anemia of any type	1.86	1.02–3.42	0.04
Age [per 1 year]	1.03	1.01–1.05	0.0008
Number of PAH-specific drug classes at 12 months (1–3)	0.97	0.7–1.35	0.86
**Third model: 3 strata risk, age, TSAT < 20% with ferritin > 100 μg/L**			
3 strata risk category	3.37	1.8–6.31	0.0001
TSAT < 20% with ferritin > 100 μg/L (regardless of hemoglobin level)	1.53	0.87–2.68	0.14
Age [per 1 year]	1.03	1.02–1.05	0.0003
**Fourth model: 3 strata risk, age and presence of ACD**			
3 strata risk category	3.46	1.8–6.66	0.0002
ACD	2.68	1.11–6.49	0.03
Age [per 1 year]	1.03	1.01–1.05	0.006
**Fifth model: 3 strata risk and presence of ACD or ACD/IDA**			
3 strata risk category	3.18	1.71–5.91	0.0003
ACD or ACD/IDA	2.14	1.1–4.41	0.03
Age [per 1 year]	1.03	1.01–1.05	0.0004

*Four-strata risk assessment available only in 12-month survivors. ACD, anemia of chronic disease; ACD/IDA, overlap of ACD and IDA; CI, confidence interval; HR, hazard ratio; IDA, iron deficiency anemia; PAH, pulmonary arterial hypertension; TSAT, transferrin saturation. All models were adjusted for European Society of Cardiology/European Respiratory Society strata mortality risk category and age.

In an extended multivariate analysis, anemia remained a significant, independent mortality risk factor after further adjustments for estimated glomerular filtration rate, PAH subtype, baseline mortality risk, and age (HR for presence of anemia: 2.5; 95% CI, 1.25–4.97; *p* = 0.009).

Additional analyses showed no significant association between mortality and deficiencies in folic acid or vitamin B12 (*p* = 0.25 and *p* = 0.8, respectively) while low serum iron levels tended to be associated with worse survival (*p* = 0.055).

## 4 Discussion

To the best of our knowledge, this is the first study to comprehensively evaluate the prognostic implications of anemia subtypes in PAH within the framework of contemporary risk stratification models. Anemia was associated with an increased calculated risk and predicted survival beyond the standard risk assessment models. Conversely, no significant associations were found between mortality and deficiencies of microelements crucial for hemoglobin metabolism, such as vitamin B12 and folic acid.

Anemia may contribute to the progression of right ventricular dysfunction via several mechanisms. Reduced hemoglobin levels impair oxygen delivery, increase myocardial workload, and exacerbate right ventricular strain. Furthermore, anemia may amplify the perception of dyspnoea, potentially inflating risk assessments based on functional parameters such as 6MWD or WHO functional class (16).

### 4.1 Iron deficiency and anemia in pulmonary arterial hypertension

Iron deficiency is a common comorbidity in PAH, affecting up to 50% of women and 28% of men with PAH (17, 18). Although iron deficiency is more prevalent in IPAH than chronic thromboembolic pulmonary hypertension (17), it is most frequently observed in CTD-PAH among the various pulmonary hypertension subtypes (19). Furthermore, in systemic sclerosis, PAH is associated with a higher prevalence of iron deficiency compared with patients without PAH (20).

Anemia has also emerged as a strong prognostic marker in PAH. In a cohort of 145 patients with PAH, hemoglobin levels were shown to strongly correlate with survival outcomes in pulmonary hypertension (21).

In another study involving 150 patients with PAH, the presence of hypochromic erythrocytes greater than 2% was identified as a strong and independent predictor of mortality and time to clinical worsening (22).

Similarly, in a cohort of 153 patients with precapillary pulmonary hypertension followed over a mean period of five years, 33% to 42% were found to have iron deficiency. These patients demonstrated significantly higher mortality compared to those without iron deficiency, irrespective of anemia (23).

Another study demonstrated that iron deficiency is associated with more severe disease and higher mortality rates in pulmonary hypertension related to chronic lung disease (24).

Our findings align with those studies, further confirming that anemia remains an independent predictor of mortality, even after adjustment for recommended risk scores and age.

### 4.2 Treatment of iron deficiency in pulmonary arterial hypertension

Despite strong observational evidence linking anemia and iron deficiency to poor outcomes, clinical trials evaluating iron supplementation in PAH have yielded mixed results.

In two randomized, double-blind, placebo-controlled, 12-week crossover studies conducted in Europe (*n* = 39) and China (*n* = 17), patients with iron deficiency received parenteral iron therapy. When analyzed separately and in combination, these studies found no significant improvement in exercise capacity, as measured by cardiopulmonary exercise testing or the 6MWD test, nor in cardiopulmonary haemodynamics, assessed by right heart catheterization, cardiac magnetic resonance imaging, or plasma NT-proBNP levels at 12 weeks (25).

Conversely, smaller studies suggest potential benefits. In a small study involving 15 patients with iron-deficient IPAH, Ruiter et al. (26) demonstrated a modest benefit in the treatment group, including improved exercise endurance time and quality of life following intravenous iron therapy.

In another study involving 20 patients with PAH, a single infusion of up to 1,000 mg of ferric carboxymaltose resulted in a net increase of 37.8 meters in 6MWD (27). Similarly, the ORION-PH trial (28) demonstrated that oral supplementation with ferric maltol was well tolerated and effective in patients with pulmonary hypertension and anemia, defined as a ferritin level of less than 100 μg/L or 100–300 μg/L with TSAT below 20% at screening. Correction of anemia was associated with improvements in right ventricular function and exercise capacity, as indicated by an increase in 6MWD.

Our findings highlight that functional iron deficiency, manifested as impaired iron availability despite preserved iron stores, was associated with adverse outcomes in PAH. This suggests that iron dysutilization may reflect an underlying inflammatory phenotype, particularly in patients with conditions such as CTD-PAH. In our cohort, CTD-PAH patients had significantly lower serum iron concentrations and more frequently presented with iron levels below the reference range, suggesting a predisposition to this abnormality. In line with ESC/ERS guidelines, we support iron repletion in all patients with anemia. In patients without anemia, iron supplementation may be considered individually, particularly in high-risk cases with evidence of inflammation or iron dysutilization. Moreover, in ACD, correction of iron metabolism may be achieved not only through supplementation but also by improving control of the underlying disease, including PAH itself.

### 4.3 Anemia mechanisms in pulmonary arterial hypertension: lessons from heart failure and experimental research

Insights from left heart failure may help elucidate the pathophysiology of anemia in PAH. Anemia is a common comorbidity in patients with left heart failure (29, 30), and several mechanisms have been proposed to explain its development. One hypothesis involves the pro-inflammatory state associated with heart failure, in which elevated levels of cytokines such as interleukin-6 contribute to anemia of chronic inflammation (31).

Additionally, chronic left heart failure has been linked to disruptions in both myelopoiesis and erythropoiesis, including reduced colony-forming potential and increased cell apoptosis (32). Hematinic deficiencies, such as reduced levels of vitamin B12, folate, and iron, have also been identified in 6, 8, and 13% of anemic patients with left heart failure, respectively. Moreover, advanced heart failure may lead to generalized gut oedema, which can impair iron absorption. Whether similar mechanisms contribute to anemia in pulmonary hypertension and its progression to right ventricular failure remains insufficiently understood (16).

Experimental models have shown that iron deficiency can elevate pulmonary artery pressure, promote right ventricular hypertrophy and vascular remodeling, and upregulate HIF1α, HIF2α, and STAT3 signaling pathways (32) — changes that are reversible with iron therapy (33). Deletion of iron regulatory protein 1 in mice induces pulmonary hypertension and polycythaemia, effects that are exacerbated by low-iron intake through HIF2α and endothelin-1 upregulation in endothelial cells (34). Interestingly, iron restriction attenuated monocrotaline-induced pulmonary hypertension without affecting serum iron levels but led to increased pulmonary arterial TfR1 expression (35). Furthermore, TfR1 heterozygous knockout mice showed reduced hypoxia-induced pulmonary hypertension and remodeling (36). Iron chelation (achieved by the administration of an iron chelator, deferoxamine) also ameliorated hypoxia-induced pulmonary hypertension and reduced oxidative stress markers in rats (37). Additionally, iron accumulation in alveolar spaces has been linked to pulmonary hypertension in idiopathic pulmonary fibrosis (38).

### 4.4 Vitamin B12 and folate: likely markers, not mediators

Vitamin B12 and folic acid deficiencies are uncommon in PAH. The reported prevalence of vitamin B12 deficiency ranges from 3% (39) to 29% (40), with the higher rate attributed to a broader definition that included elevated methylmalonic acid levels despite normal or borderline serum vitamin B12 levels. Folic acid insufficiency appears to be even rarer (39).

Similarly, in our study, deficiencies in vitamin B12 and folic acid were uncommon, and supplementation was rare. Interestingly, higher plasma levels of B12 and folic acid were observed in patients with anemia and those at higher risk. Given the minimal supplementation and low prevalence of true deficiencies, these findings likely reflect inflammatory activation, impaired vitamin clearance, or altered metabolism rather than adequate repletion. This supports the notion that the primary contributors to anemia in PAH are disturbances in iron metabolism rather than hematinic deficiencies.

### 4.5 Strengths and limitations

This study has several strengths, including a comprehensive analysis of anemia and iron-related parameters in PAH, detailed differentiation of anemia subtypes, and long-term follow-up, which enhance the reliability of the findings. However, limitations include the single-center design and a relatively small sample size. Not all blood samples were obtained strictly at the time of diagnosis, and although supplementation data are provided in Supplementary Table 1, incomplete information on pre-sampling treatment introduces a risk of reverse causality. Iron status and related parameters (including ferritin, TSAT, serum iron, folate, and vitamin B12) were assessed only once, using frozen baseline serum samples collected at diagnosis and analyzed later.

## 5 Conclusion

Anemia and disturbances in iron metabolism are common in PAH, whereas vitamin B12 and folate deficiencies are relatively rare. Anemia, particularly ACD and ACD/IDA overlap, was associated with a lower likelihood of clinical improvement and with higher long-term mortality, independent of ESC/ERS risk scores. These findings indicate that specific anemia subtypes provide prognostic information beyond current risk models and should be considered for inclusion in future PAH risk stratification frameworks. Further studies are warranted to validate these observations and to determine whether correction of distinct anemia phenotypes improves clinical outcomes.

## Data Availability

The raw data supporting the conclusions of this article will be made available by the authors, without undue reservation.
