# Supplementary material for: Clinical and prognostic significance of various anemia types in pulmonary arterial hypertension
Source: Front Med (Lausanne). 2025 Dec 10;12:1728165. doi: 10.3389/fmed.2025.1728165 (PMC12727604; doi:10.3389/fmed.2025.1728165)
Supplement: Supplementary file 1 [file Supplementary_file_1.docx]

**Supplementary results**

Characterization of ACD subgroup

Among the patients classified as having ACD, the predominant features were consistent with a chronic inflammatory and multimorbid phenotype. The median age in this group was 69 years (range: 67–76). Chronic kidney disease, defined as eGFR <60 mL/min/1.73 m², was present in 71.4% of patients. Cardiometabolic comorbidities were frequent, including arterial hypertension in 71.4%, type 2 diabetes mellitus in 42.9%, and coronary artery disease in 85.7%. Folate deficiency was observed in 28.6% of patients, while no cases of vitamin B12 deficiency or connective tissue disease were identified. These features are consistent with the clinical context of ACD, where renal impairment, chronic inflammation, and age-related factors likely contribute to iron dysutilization and anaemia.

Characterization of multifactorial anaemia

Among the nine patients classified as having multifactorial anaemia (TSAT >20%, not meeting criteria for other anaemia subtypes), a variety of contributing factors were identified. The most common was chronic kidney disease, with six patients (66.7%) meeting the KDIGO (Kidney Disease: Improving Global Outcomes) definition, i.e., an estimated glomerular filtration rate <60 mL/min/1.73 m² sustained for ≥3 months. One patient was on chronic dialysis. Additionally, four patients had folate deficiency, two presented with bone marrow abnormalities (polycythemia vera and hemolytic anaemia with thrombocytosis), and two had connective tissue diseases (rheumatoid arthritis and systemic sclerosis), which may reflect chronic inflammation or malabsorption. These overlapping mechanisms support the classification of this subgroup as multifactorial.

Characterization of ACD/IDA anaemia

Among the six patients classified as having ACD/IDA overlap (TSAT <20% with ferritin 30–100 µg/L), the clinical profile reflected a mixed phenotype of iron restriction and comorbidity burden. Chronic kidney disease, defined as eGFR <60 mL/min/1.73 m², was present in 33.3% of patients, type 2 diabetes mellitus in 50.0%, arterial hypertension in 16.7%, coronary artery disease, one patient had thrombocytosis. Folate deficiency was identified in 33.3% of patients, while vitamin B12 deficiency was less common (16.7%). These features are consistent with the overlap phenotype, where impaired iron availability coexists with multiple comorbid conditions, nutritional deficiencies, and, in some cases, underlying marrow dysfunction.

| Supplementation: | Whole population | Anemia | No anemia | p |
| --- | --- | --- | --- | --- |
| Iron at the time of blood collection [n,%] | 10 (7.9%) | 4 (18.2%) | 6 (5.7%) | 0.12^⊛^ |
| Iron within 12 months [n,%] | 8 (7.6%) | 2 (9.1%) | 6 (5.7%) | 0.55 |
| Folic acid at the time of blood collection [n,%] | 7 (5.5%) | 4 (18.2%) | 3 (2.9%) | 0.02 ^⊛^ |
| Folic acid within 12 months [n,%] | 1 (0.8%) | 0 | 1 (1%) | 0.65 |
| B12 at the time of blood collection [n,%] | 3 (2.4%) | 2 (9.1%) | 1 (1%) | 0.12 ^⊛^ |
| B12 within 12 months [n,%] | 0 | 0 | 0 | - |
| ⊛ Yates correction was applied |  |  |  |  |

Supplementary Table 1. Comparison of oral supplementation with iron, folic acid, or vitamin B12 at baseline and during follow-up.

Supplementary Table 2. Comparison of patients’ baseline characteristics stratified by clinical risk category.

|  | Whole population | Low mortality risk | Intermediate mortality risk | High mortality risk | p |
| --- | --- | --- | --- | --- | --- |
| N | 127 | 14 | 99 | 14 |  |
| Quantitative parameters |  |  |  |  |  |
| TSAT <20% with ferritin >100 μg/L | 22 (17.3%) | 1 (7.1%) | 18 (18.2%) | 3 (21.4%) | 0.54 |
| Anaemia categories |  |  |  |  |  |
| ACD | 7 (5.5%) | 0 | 4 (4%) | 3 (21.4%) | 0.018 |
| ACD or ACD/IDA | 13 (10.2%) | 0 | 10 (10.1%) | 3 (21.4%) | 0.17 |
| IDA | 0 | 0 | 0 | 0 | - |
| ID (by NEJM) | 42 (33.1%) | 1 (7.1%) | 34 (34.3%) | 7 (50%) | 0.047 |
| ID (by ESC guidelines) | 71 (55.9%) | 7 (50%) | 55 (55.6%) | 9 (64%) | 0.74 |
| Morphology |  |  |  |  |  |
| Hemoglobin < LRL [n,%] | 22 (17.3%) | 2 (14.3%) | 16 (16.2%) | 4 (28.6%) | 0.49 |
| RBC [10^6/ul] | 4.84 (4.42;5.29) | 4.5 (4.39;5.09) | 4.86 (4.46;5.37) | 4.64 (4.3;5.3) | 0.32 |
| HGB [g/dL] | 14.2 (12.9;15.6) | 13.2 (12.6;15.5) | 14.3 (13;15.6) | 13.5 (12.1;15.6) | 0.22 |
| HCT [%] | 42.2 (39.4;46.8) | 39.4 (38.2;43.1) | 42.6 (40;47) | 42.1 (39.2;46.8) | 0.13 |
| MCV [fL] | 89.1 (84.2;92.2) | 88 (84.2;90.4) | 89.2 (84.2;92.2) | 89.5 (84.7;92.9) | 0.73 |
| MCH [pg] | 29.5 (27.7;30.9) | 29.8 (27.7;30.5) | 29.5 (28;31.1) | 29.6 (27.5;30.3) | 0.89 |
| MCHC [g/dL] | 33.2 (32.3;34.1) | 33.8 (32.5;34.5) | 33.2 (32.3;34.1) | 32.9 (31.3;33.3) | 0.14 |
| RDW [%] | 15 (13.8;16.5) | 13.9 (13.5;15.2) | 15 (13.7;16.6) | 15.5 (14.5;16.6) | 0.11 |
| PLT [10^3/ul] | 199 (161;265) | 264 (168;311) | 195 (162;252) | 194 (156;305) | 0.4 |
| PDW [fL] | 13 (11.5;14.8) | 13.1 (11.5;13.8) | 13 (11.5;15) | 12.3 (11.4;14.7) | 0.91 |
| MPV [fL] | 10.9 (10.2;11.6) | 10.9 (10.2;11.3) | 10.8 (10.2;11.7) | 11.1 (10.5;11.8) | 0.84 |
| PCT [%] | 0.22 (0.19;0.27) | 0.22 (0.2;0.33) | 0.22 (0.19;0.27) | 0.23 (0.17;0.34) | 0.47 |
| Immunochemistry |  |  |  |  |  |
| Folic acid [ng/ml] | 4.8 (3.6;7.3) | 5,4 (3.8;7.5) | 4,8 (3.7;7.7) | 4,7 (3.1;6) | 0.69 |
| Folic acid < LRL [n,%] | 35 (27.6%) | 4 (28,6%) | 26 (26,3%) | 5 (35,7%) | 0.76 |
| B12 [pg/ml] | 459 (339;591) | 450 (350;534) | 445 (330;591) | 506 (448;1122) | 0.24 |
| B12 < LRL [N,%] | 5 (3.9%) | 1 (7.1%) | 3 (3.03%) | 1 (7.1%) | 0.61 |
| Biochemistry |  |  |  |  |  |
| TIBC [umol/L] | 60.3 (51.1;68.2) | 57.6 (49.9;64.9) | 60.3 (51.5;67.8) | 60.8 (49.6;74.3) | 0.75 |
| TIBC < LRL [N,%] | 12 (6.5%) | 1 (7.1%) | 9 (9.1%) | 2 (14.3%) | 0.78 |
| TIBC >URL [N,%] | 26 (20.5%) | 2 (14.3%) | 20 (20.2%) | 4 (28.6%) | 0.64 |
| UIBC [umol/L] | 44.5 (35.3;54.1) | 38.8 (33.5;48.7) | 44.5 (35.3;54) | 52,3 (39.6;61.2) | 0.11 |
| UIBC < LRL [N,%] | 5 (3.9%) | 1 (7.1%) | 4 (4%) | 0 | 0.62 |
| UIBC > URL [N,%] | 14 (11%) | 0 | 11 (11.1%) | 3 (21.4%) | 0.19 |
| Transferrin saturation [%] | 24 (15;33) | 29.5 (21;36) | 25 (15;33) † | 17.5 (11;22) ǂǂ | 0.005 |
| Transferrin saturation < LRL [N,%] | 54 (42.5%) | 1 (7.1%) | 44 (44.4%) * | 9 (64.3%) ǂ | 0.007 |
| iron [umol/L] | 13.6 (9;19.9) | 16.5 (13.3;21.9) | 14.1 (8.6;20) † | 9.7 (7.6;11.1) ǂǂ | 0.01 |
| iron < LRL [N,%] | 52 (40.9%) | 3 (21.4%) | 39 (39.4%) † | 10 (71.4%) ǂǂ | 0.02 |
| ferritin [ug/L] | 104.1 (67.5;237.1) | 95 (80.1;291) | 108.6 (70;234) | 95.5 (36.8;149.1) | 0.56 |
| ferritin < LRL [N,%] | 11 (8.7%) | 2 (14.3%) | 7 (7.1%) | 2 (14.3%) | 0.49 |
| ferritin > URL [N,%] | 10 (7.9%) | 2 (14.3%) | 7 (7.1%) | 1 (7.1%) | 0.64 |

⊛ Yates correction was applied

* p<0.05 between group with intermediate and low mortality risk (post hoc)

** p<0.001 between group with intermediate and low mortality risk (post hoc)

† p<0.05 between group with intermediate and high mortality risk (post hoc)

†† p<0.001 between group with intermediate and high mortality risk (post hoc)

ǂ p<0.05 between group with high and low mortality risk (post hoc)

ǂǂ p<0.001 between group with high and low mortality risk (post hoc)

Supplementary Table 3. Comparison of baseline characteristics between patients with idiopathic pulmonary arterial hypertension and those with pulmonary arterial hypertension associated with connective tissue disease.

|  | Idiopathic pulmonary arterial hypertension | Pulmonary arterial hypertension associated with connective tissue disease | P |
| --- | --- | --- | --- |
|  | 100 | 27 |  |
| Age [years] | 56 (43;69) | 66 (52;71) | 0.09 |
| Sex [female, n,%] | 65 (65%) | 24 (88.9%) | 0.03 ⊛ |
| Baseline clinical risk in PAH |  |  |  |
| Low | 13 (13%) | 1 (3.7%) | 0.12 |
| Intermediate | 74 (74%) | 25 (92.6%) |  |
| High | 13 (13%) | 1 (3.7%) |  |
| Iron deficiency | 56 (56%) | 15 (55.6%) | 0.97 |
| Dysutilization of iron | 16 (16%) | 6 (22.2%) | 0.08 |
| Anemia |  |  |  |
| Any type of anaemia [n,%] | 16 (16%) | 6 (22.2%) | 0.08 |
| ACD [n,%] | 6 (6%) | 1 (3.7%) | 0.99⊛ |
| overlap ACD/IDA [n,%] | 3 (3%) | 3 (11.1%) | 0.21⊛ |
| Multifactorial anemia | 7 (7%) | 2 (7.4%) | 0.73⊛ |
| ACD or ACD/IDA [n,%] | 9 (9%) | 4 (14.8%) | 0.84⊛ |
| Isolated IDA [n,%] | 0 | 0 | - |
| Folic acid and B12 |  |  |  |
| Folic acid [ng/ml] | 4.9 (3.9 ; 7.3) | 4.5 (3.2 ; 8.5) | 0.53 |
| Folic acid < LRL [n,%] | 24 (24%) | 11 (40.7%) | 0.08 |
| B12 [pg/ml] | 459 (335 ; 597.5) | 452 (341 ; 587) | 0.66 |
| B12 < LRL [n,%] | 4 (4%) | 1 (3.7%) | 0.63⊛ |
| iron metabolism parameters |  |  |  |
| TIBC [µmol/L] | 60.7 (53 ; 68.2) | 52.3 (43.5 ; 65.4) | 0.03 |
| TIBC < LRL [n,%] | 4 (4%) | 9 (29.6%) | 0.0002 |
| TIBC >URL [n,%] | 21 (21%) | 8 (18.5%) | 0.78 |
| UIBC [µmol/L] | 46.6 (36.8 ; 54.3) | 38 (33.3 ; 54) | 0.16 |
| UIBC < LRL [n,%] | 4 (4%) | 1 (3.7%) | 0.63 |
| UIBC > URL [n,%] | 10 (10%) | 4 (14.8%) | 0.72 |
| Transferrin saturation [%] | 25 (17 ; 33) | 22 (13 ; 28) | 0.17 |
| Transferrin saturation < LRL [n,%] | 41 (41%) | 13 (48.5%) | 0.51 |
| iron [µmol/L] | 15.5 (9.5 ; 20.2) | 9.7 (6.6 ; 14.4) | 0.02 |
| iron < LRL [n,%] | 36 (36%) | 16 (59.3%) | 0.03 |
| ferritin [ug/L] | 103.2 (65.5 ; 208.8) | 134.4 (75 ; 259.2) | 0.33 |
| ferritin < LRL [n,%] | 9 (9%) | 2 (7.4%) | 0.9⊛ |
| ferritin > URL [n,%] | 9 (9%) | 1 (3.7%) | 0.61⊛ |

⊛ Yates correction was applied


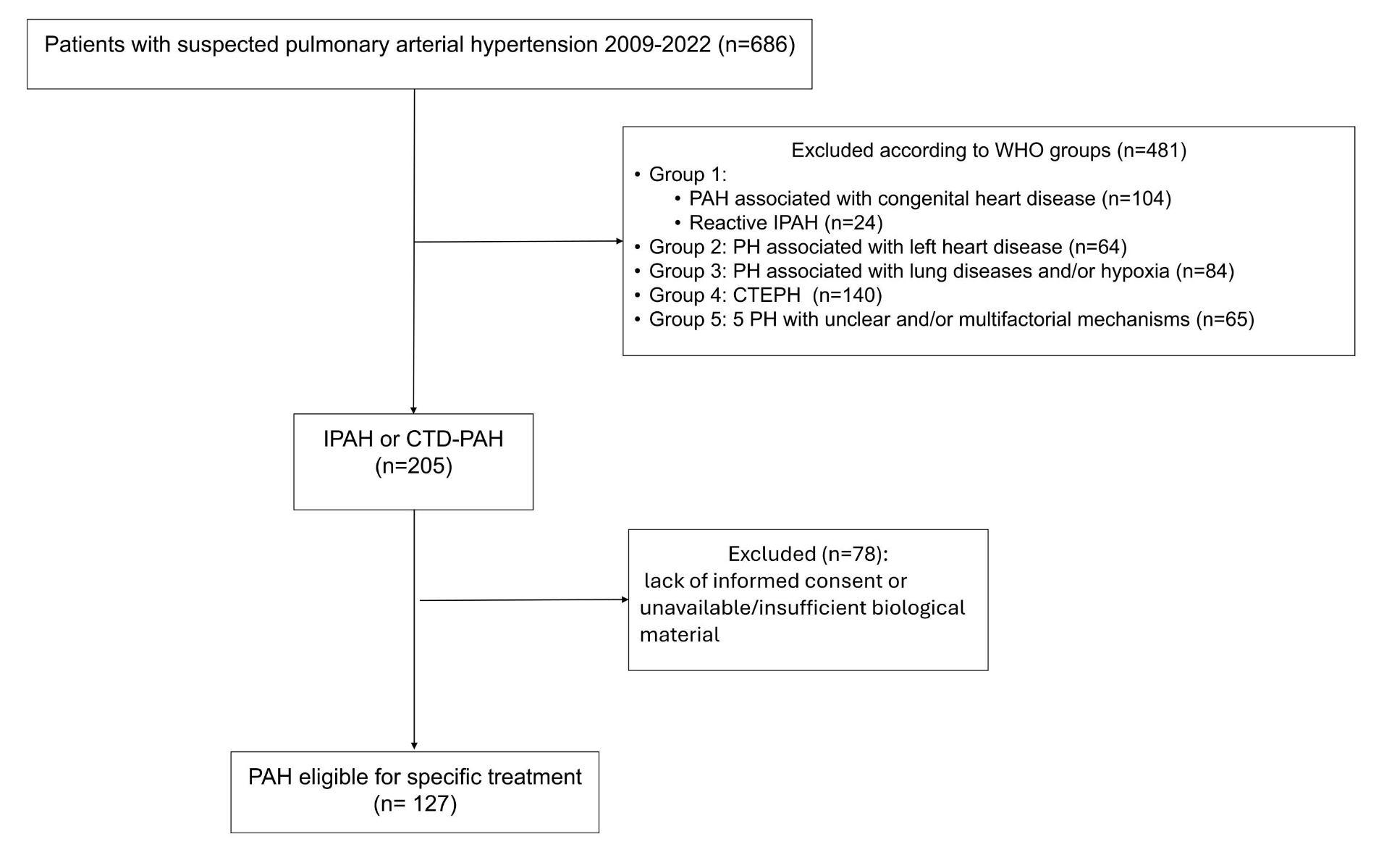


Supplementary Figure 1. Flowchart illustrating the process of patient inclusion and exclusion.
